# Structural insights into the substrate specificity of IMP-6 and IMP-1 metallo-β-lactamases

**DOI:** 10.1093/jb/mvac080

**Published:** 2022-09-29

**Authors:** Keizo Yamamoto, Hideaki Tanaka, Genji Kurisu, Ryuichi Nakano, Hisakazu Yano, Hiromi Sakai

**Affiliations:** Department of Chemistry, Nara Medical University, 840 Shojo-Cho, Kashihara, Nara 634-8521, Japan; Institute for Protein Research, Osaka University, 3-2 Yamadaoka, Suita, Osaka 565-0871, Japan; Institute for Protein Research, Osaka University, 3-2 Yamadaoka, Suita, Osaka 565-0871, Japan; Department of Microbiology and Infectious Diseases, Nara Medical University, 840 Shojo-Cho, Kashihara, Nara 634-8521, Japan; Department of Microbiology and Infectious Diseases, Nara Medical University, 840 Shojo-Cho, Kashihara, Nara 634-8521, Japan; Department of Chemistry, Nara Medical University, 840 Shojo-Cho, Kashihara, Nara 634-8521, Japan

**Keywords:** carbapenemase, X-ray crystallography, substrate specificity, metallo-β-lactamase, loop flexibility

## Abstract

IMP-type metallo-β-lactamases confer resistance to carbapenems and a broad spectrum of β-lactam antibiotics. IMP-6 and IMP-1 differ by only a point mutation: Ser262 in IMP-1 and Gly262 in IMP-6. The *k*_cat_/*K*_m_ values of IMP-1 for imipenem and meropenem are nearly identical; however, for IMP-6, the *k*_cat_/*K*_m_ for meropenem is 7-fold that for imipenem. In clinical practice, this may result in an ineffective therapeutic regimen and, consequently, in treatment failure. Here, we report the crystal structures of IMP-6 and IMP-1 with the same space group and similar cell constants at resolutions of 1.70 and 1.94 Å, respectively. The overall structures of IMP-6 and IMP-1 are similar. However, the loop region (residues 60–66), which participates in substrate binding, is more flexible in IMP-6 than in IMP-1. This difference in flexibility determines the substrate specificity of IMP-type metallo-β-lactamases for imipenem and meropenem. The amino acid at position 262 alters the mobility of His263; this affects the flexibility of the loop via a hydrogen bond with Pro68, which plays the role of a hinge in IMP-type metallo-β-lactamases. The substitution of Pro68 with a glycine elicited an increase in the *K*_m_ of IMP-6 for imipenem, whereas the affinity for meropenem remained unchanged.

## Graphical Abstract

**Graphical Abstract:**
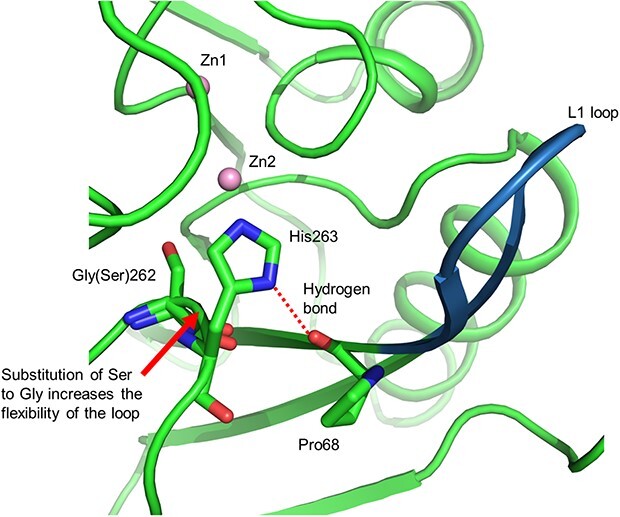

## Abbreviations

BcII: *Bacillus cereus* metallo-β-lactamase
GES: Guiana extended-spectrum β-lactamase
IMP: imipenemase
KPC: *Klebsiella pneumoniae* carbapenemase
MBL: metallo-β-lactamase
NDM: New Delhi metallo-β-lactamase
OXA: oxacillin resistant β-lactamase
VIM: Verona Integron-encoded metallo-β-lactamase

Carbapenems, such as imipenem and meropenem, are often considered ‘last resort’ drugs for the treatment of severe infections due to Gram-negative pathogens. The emergence and spread of carbapenem-resistant *Enterobacteriaceae* have aroused a growing concern (*1**,**2*), as these bacteria express carbapenemases, a family of β-lactamases that hydrolyse the β-lactam ring of carbapenems to inactivate them.

The Ambler classification divides β-lactamases into four classes (*3*), of which three (A, B and D) include carbapenemases. Class A carbapenemases include *Klebsiella pneumoniae* carbapenemase (KPC) (*4*) and some variants of Guiana extended-spectrum β-lactamases (GES) (*5*). Class B carbapenemases, which harbour two catalytic zinc ions, comprise metallo-β-lactamases (MBL) and include New Delhi metallo-β-lactamases (NDM) (*6*), imipenemases (IMP) (*7*) and Verona Integron-encoded MBL(VIM) (*8*). The reaction mechanism of NDM-1 and related metallo-β-lactamases have been previously postulated (*9**,**10*). Class D carbapenemases include oxacillin-resistant β-lactamases, OXA-48 type β-lactamases and their variants (*11*). KPC- and OXA-48-like variants are predominant in Europe and the United States of America, whereas KPC and NDM are predominantly found in China (*12*).

To date, at least 96 variants of IMP-type MBLs have been deposited (https://www.ncbi.nlm.nih.gov/genbank/). In Japan, clinical isolates of carbapenemase-producing *Enterobacteriaceae* mainly produce IMP-type carbapenemases (EC 3.5.2.6), especially IMP-1 and IMP-6 (*13–15*).

MBL IMP-1 was originally isolated from *Serratia marcescens* in Japan in 1991 (*7*), and later from *Pseudomonas aeruginosa* (*16*). Yano *et al.* reported the first isolate of IMP-6-producing *S. marcescens* found in the urine of a Japanese patient with a urinary tract infection (*17*). The *bla*_IMP-1_ and *bla*_IMP-6_ genes differ by a single-point mutation where the adenine base at nucleotide 640 in *bla*_IMP-1_ is replaced by guanine. This point mutation results in an amino acid substitution at position 262 of the protein sequence, resulting in a Ser262 in IMP-1 and a Gly262 in IMP-6.

The substrate specificities of wild-type IMP-1 and mutant IMP-1 S262G have been systematically compared. Two antibiotics, cephalothin and cefotaxime, are efficiently hydrolysed by IMP-1 regardless of the amino acid substitution, whereas hydrolysis of cephaloridine, ceftazidime, ampicillin, benzylpenicillin and imipenem by IMP-1 S262G is less efficient (*18*). To explain these observations, Oelschlaeger *et al.* proposed a ‘domino effect’ using molecular dynamics simulations and mutational experiments (*19–22*). The effect of this amino acid substitution has also been investigated in the structurally similar *Bacillus cereus* metallo-β-lactamase (BcII) (*23**,**24*). Moreover, IMP-1 and IMP-6 have different substrate specificities for carbapenems. The *k*_cat_/*K*_m_ ratio of IMP-1 for meropenem and imipenem are almost identical (*25**,**26*), whereas the *k*_cat_/*K*_m_ ratio of IMP-6 for meropenem is seven times higher than that for imipenem (*17*). Thus, isolates producing IMP-6 may be erroneously categorized as imipenem-susceptible, which may result in treatment failure in patients (*27*).

Substrate and inhibitor binding to the active site of MBL has been structurally characterized in different MBLs. Concha *et al.* reported the crystal structure of *P. aeruginosa* IMP-1 (PDB ID 1DDK) and its complex with a mercaptocarboxylate inhibitor (PDB ID 1DD6) in 2000 (*28*). IMP-1 has an αβ/βα fold that is conserved in MBL such as the L1 MBL from *Stenotrophomonas maltophilia* (PDB ID 1AML) (*29*). The active site that is located at the interface of the two αβ units harbours two zinc ions and is covered by a mobile loop connecting β2 and β3. This loop, termed L1 in this study, adopts an open conformation in the inhibitor-free protein and a closed conformation in the inhibitor-bound protein (*28*). While the structure of the active site, especially that of the loop region, is thought to dictate the differences in substrate specificity, recent structures suggest otherwise. The structure of *S. marcescens* IMP-1 (PDB ID 5EV6) shows both open/closed structures of the mobile loop (L1) in different molecules in the asymmetric unit (*30*). Similarly, two structures of *P. aeruginosa* IMP-13 show the mobile loop (L1) in both the open (PDB ID 6R79) and closed (PDB ID 6R78) conformations (*31*). Thus, the structure of the L1 loop region may depend on the crystal packing.

Here, to elucidate the structural differences in substrate specificities between IMP-6 and IMP-1, especially the differences in their substrate specificities towards meropenem and imipenem, we report the structure of IMP-6 that was experimentally determined at a resolution of 1.70 Å. To minimize the influence of crystal packing, a structural comparison was performed using a crystal structure of IMP-1 at a resolution of 1.94 Å obtained from a crystal isomorphous to those of IMP-6. We discuss the relationship between the flexibility in the L1 loop and the differences in affinities of IMP-6 and IMP-1 for imipenem and meropenem. Furthermore, we analyse the relationship of Pro68 with Gly262 in IMP-6/Ser262 in IMP-1 using P68G mutants of IMP-6 and IMP-1.

## Materials and Methods

### *Cloning of bla*_IMP-1_ and *bla*_IMP-6_ genes

The starting plasmids were obtained from the clinical isolates *Escherichia coli* 58–132 and NR390 maintained at the Nara Medical University Hospital (Kashihara, Nara, Japan). The *bla*_IMP-1_ and *bla*_IMP-6_ genes were amplified by PCR using primers IMP-6-full-F (5′-AGCAAGTTATCTGTATATATTTTTGTTTTG-3′) and IMP-6-mat-R-Bam (5′-ATATAGGATCCTTAGTGGTTTTGATGGTTT-3′). PCR products were digested with the restriction enzyme *Bam* HI (TaKaRa Bio, Shiga, Japan). A pET28a vector (Merck Millipore, Darmstadt, Germany) was digested with *Nco* I, blunted with a blunting kit (TaKaRa Bio, Shiga, Japan) and further digested with *Bam* HI. The vector and the inserts *bla*_IMP-6_ and *bla*_IMP-1_ were ligated using T4 ligase to generate the pET28a-imp6 and pET28a-imp1 plasmids, respectively. *Escherichia coli* BL21(DE3) cells were transformed with each plasmid.

### Expression and purification of recombinant IMP-6 and IMP-1

*Escherichia coli* transformants carrying *bla*_IMP-6_ or *bla*_IMP-1_ were inoculated into a Luria-Bertani medium supplemented with 30 μg ml^−1^ kanamycin and incubated at 310 K. Isopropyl thio-β-d-galactoside (final concentration: 0.02 mM) was added when the optical density at 600 nm reached 0.5, and the culture was further incubated for 20 h at 293 K. Cells were resuspended in buffer A (20 mM Tris/HCl (pH 7.5), 50 μM ZnSO_4_) and sonicated. The cell-free extract was applied to a Macro-Prep High S Support column (Bio-Rad Laboratories, Inc., Hercules, CA, USA) and washed with buffer A. The enzymes were eluted with buffer A containing 0.3 M NaCl. Fractions containing IMP-6 or IMP-1 were pooled, dialysed against buffer A and injected into a CM-Toyopearl 650S column (Tosoh, Tokyo, Japan) equilibrated with buffer A. The enzymes were eluted with a linear gradient of NaCl (0–0.25 M) in the same buffer. Analysis of the N-terminal amino acid sequence of IMP-6 confirmed that the signal sequence was removed in the host cells.

### Protein crystallization and crystallographic data collection

Prior to crystallization, IMP-6 and IMP-1 were dialysed separately against 5 mM HEPES buffer (pH 7.3) with 50 μM ZnSO_4_ and concentrated to 15 mg ml^−1^. IMP-6 drops were prepared by adding 3 μl protein solution and 3 μl reservoir solution containing 0.1 M sodium acetate (pH 4.6), 0.2 M ammonium acetate and 17% (w/v) polyethylene glycol 8000; these drops were equilibrated over 0.5 ml reservoir solution using the hanging-drop vapour-diffusion method at 288 K. IMP-1 drops were prepared similarly, but the reservoir solution contained 0.1 M sodium acetate (pH 5.0), 0.2 M ammonium acetate and 22% (w/v) polyethylene glycol 8000. Rod-shaped crystals grew to a maximum dimension of 0.1 × 0.1 × 0.2 mm in 2 weeks. IMP-6 and IMP-1 crystals were flash-frozen in liquid nitrogen using the respective reservoir solution containing 10% (v/v) glycerol as a cryoprotectant. X-ray diffraction data were collected at 100 K on beamline BL44XU at SPring-8 (Hyogo, Japan) using an Eiger X 16 M detector (Dectris, Philadelphia, PA, USA). Data were processed using XDS (*32*).

### Structure determination and refinement

The structures were solved by the molecular replacement using the Molrep (*33*) program from the CCP4 program suite (*34*) and the coordinates of *P. aeruginosa* IMP-1 (PDB ID 1DDK) as the search model. Manual model building and refinement were carried out using Coot (*35*) and Refmac5 (*36*). Molecular graphic images were prepared using PyMOL (Schrödinger, LLC, New York, NY, USA). Standard MBL amino acid numbering was used (*37*).

**Table 1:** Data collection and refinement statistics

	IMP-6	IMP-1
Data collection		
Space group	*P*2_1_2_1_2_1_	*P*2_1_2_1_2_1_
*a* (Å)	49.156	49.321
*b* (Å)	78.340	78.332
*c* (Å)	260.225	259.945
Resolution range ^a^ (Å)	50.00–1.70 (1.79–1.70)	50.00–1.94 (2.06–1.94)
Observed reflections ^a^	758,783 (124620)	497,008 (76149)
Unique reflections ^a^	112,203 (17827)	75,269 (11934)
Completeness ^a^ (%)	100.0 (99.9)	99.8 (98.9)
Redundancy ^a^	6.76 (6.99)	6.60 (6.38)
Average I/σ^a^	13.73 (2.46)	6.89 (1.05)
CC_1/2_	0.997 (0.941)	0.993 (0.937)
*R*_merge_ ^a^ ^,^ ^b^ (%)	0.069 (0.512)	0.124 (0.783)
Refinement		
Resolution limit (Å)	48.3–1.70	46.1–1.94
*R*_work_ ^c^/*R*_free_^d^	0.205/0.238	0.249/0.292
No. of protein atoms	6756	6764
No. of water molecules	377	128
No. of zinc ions	8	8
RMSD		
Bond length (Å)	0.010	0.075
Bond angle (°)	1.569	1.43
Average B factor (Å^2^)		
Main chain	37.5	53.0
Side chain	43.8	59.8
Water molecules	39.0	44.9
Ramachandran plot statistics (%)		
Most favoured	96.5	95.7
Allowed	2.3	3.1
Outliers	1.2	1.2

a Values for the highest resolution shells are given in parentheses.

b *R*_merge_ = Σ*_hkl_*Σ*_i_*|*I*(*hkl*)*_i_*-‹*I*(*hkl*)›|/Σ*_hkl_I*(*hkl*).

c *R*_work_ = Σ(*F*_obs_-*F*_calc_)/Σ(*F*_obs_).

d *R*_free_: crystallographic *R*-factor based on 5% of the data withheld from the refinement for cross-validation.

### Docking simulation between IMP-6 and hydrolysed substrates

IMP-13 in complex with hydrolysed imipenem (PDB ID 6RZS) or IMP-13 in complex with hydrolysed meropenem (PDB ID 6RZR) was superposed on IMP-6. Docking simulations between IMP-6 and hydrolysed substrates were performed using the MF myPresto ver. 3.2 mmMPApp2 application (FiatLux Co. Ltd., Tokyo, Japan).

### Preparation of the P68G mutants of IMP-6 and IMP-1

The P68G mutants of IMP-6 and IMP-1 were constructed by site-directed mutagenesis using a KOD plus mutagenesis kit (TOYOBO Co., Osaka, Japan). Plasmids pET28a-imp6 or pET28a-imp1were used as templates. The following oligonucleotide primers were used: P50Gfwd (5′-GGGTGGGGCGTTGTTGGTAAACATGGTTTGGTGG-3′) and P50Grev (5′-CCACCAAACCATGTTTACCAACAACGCCCCACCC-3′). The introduced mutation was confirmed by DNA sequencing. The mutant enzymes were expressed and purified using the same method as that of the wild-type enzymes.

### Measurement of kinetic constants of P68G mutants

Enzyme activities were determined by spectrophotometry (V-730 BIO, JASCO, Tokyo, Japan) at 303 K in 20 mM HEPES buffer (pH 7.0) with 50 μM ZnSO_4_. The wavelengths and extinction coefficients used in this study were the same as those reported by Laraki *et al.* (*26*). Protein concentrations were determined using a BCA Protein Assay Kit (PIERCE, Illinois, USA) and bovine serum albumin as the standard. The enzyme was diluted with the assay buffer containing 20 μg mL^−1^ bovine serum albumin (BSA) to prevent denaturation. The values of the kinetic parameters (*K_m_* and *k*_cat_) were obtained by a double-reciprocal (Lineweaver-Burk) plot of initial steady-state velocities at different substrate concentrations (*38*).

## Results and Discussion

### Quality of the model

The initial structures of IMP-6 and IMP-1 were solved using the coordinates of *P. aeruginosa* IMP-1 as the molecular replacement template. The structure of IMP-6 was refined to a crystallographic *R*-factor of 20.8% (*R*_free_ = 23.3%) for 112,203 unique reflections in the resolution range from 48.3 to 1.70 Å. The structure of IMP-1 was refined to a crystallographic *R*-factor of 25.8% (*R*_free_ = 28.7%) for 75,259 unique reflections in the resolution range from 46.1 to 1.94 Å. Table 1 summarizes data collection and refinement statistics. The refinement of IMP-1 ends with a high R-factor; the crystals of IMP-1 are less crystalline than those of IMP-6. This is demonstrated by the fact that the average I/σ and *R*_merge_ of IMP-1 are worse than those of IMP-6 (Table 1). Insufficient data, especially for the outermost shell, may be the reason for why IMP-1 exhibits a higher R-factor during refinement. Both enzymes were crystallized in the same space group and with similar cell constants. The asymmetric units of both crystals contained four identical protein molecules (chains A, B, C and D) and eight zinc ions in the four identical active sites. Therefore, these structures enable the direct comparison of IMP-6 and IMP-1 structures without considering the influence of crystallographic packing. Analysis of the main-chain torsion angles of all the residues showed that 96.5% and 95.7% of residues in IMP-6 and IMP-1, respectively, are located in the most favoured regions of the Ramachandran plot; 2.3% and 3.1% are located in the allowed regions, respectively. The average B factor of IMP-1 is higher than that of IMP-6 (Table 1). The B factor is an indicator of flexibility; it represents the degree of fluctuation of an atom in the crystal and tends to be higher with lower resolution in a structural analysis. However, by comparing the average B factor of the L1 portion with the average B factor of the other portions, it would be possible to compare the L1 flexibility of IMP-6 and IMP-1.

**Fig. 1:**
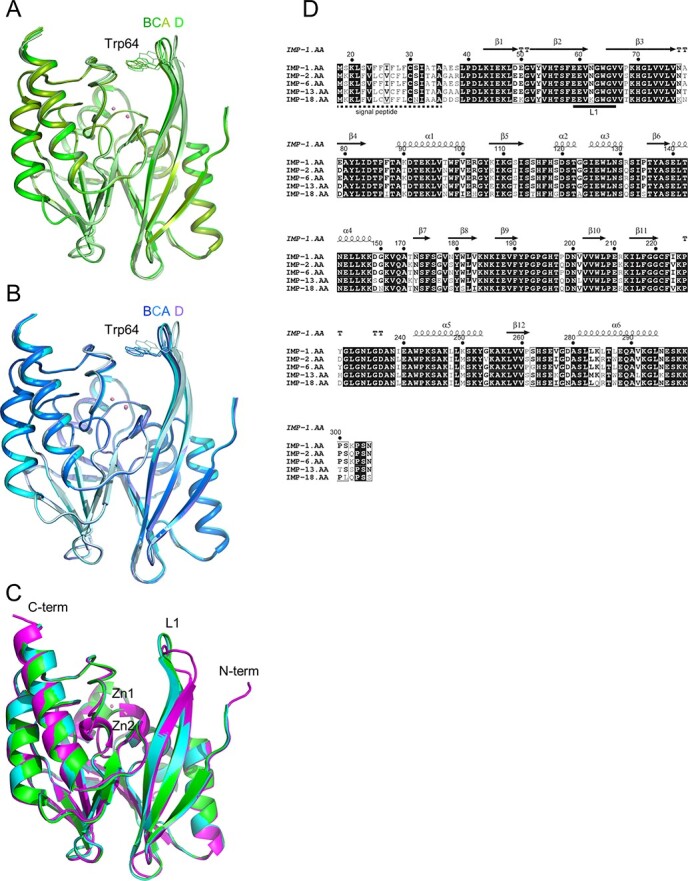
**Superposition of IMP-6 and IMP-1 metallo-β-lactamase.** (A) Superposition of chains A, B, C and D of IMP-6, shown in green, split pea, lime and pale green, respectively. (B) Superposition of chains A, B, C and D of IMP-1, shown in cyan, marine, teal and pale cyan, respectively. (C) Superposition of chain A of IMP-6, IMP-1 and IMP-1 (open conformation) (PDB ID 1DDK). IMP-6 and IMP-1 are coloured in green and cyan, respectively. Two zinc ions are represented as pink spheres. IMP-1 (PDB ID 1DDK) is coloured in magenta. (D) The structure-based sequence alignment of IMP-6 and IMP-1 with other structure-solved IMP-type metallo-β-lactamases using the structure of IMP-1 from this study. References for each sequence are as follows: IMP-1, S71932; IMP-2, AB182996; IMP-6, AB753460; IMP-13, AJ512502; and IMP-18, AY780674. The figure was produced using ESPript 3.0 program (http://espript.ibcp.fr) (49).

### Overall structure of IMP-6 and structural comparison of IMP-1 and IMP-6

The overall root mean square deviations (RMSD) for the main-chain atoms between the chains A, B, C and D of IMP-6 were all less than 0.50 Å, indicating that the four protein molecules are nearly identical (Fig. 1A). Although the four molecules in the crystallographic asymmetric unit showed the identical main chain structures, the comparison revealed that only the Trp64 of chain D located at the interface with a crystallographic symmetry mate exhibited unique side-chain conformation. In chains A, B and C, the conformation of the Trp64 side chain similarly interacted with each other in the asymmetric unit. Identical features were seen in the crystal structure of IMP-1 (Fig. 1B). Thus, hereafter, we focus our discussion on the structures of chains A of IMP-6 and IMP-1.

The overall structure of IMP-6 adopts an αβ/βα sandwich structure, typical of class B1 MBLs (*37*), such as IMP-1 (*28*), NDM-1 (*39*), CcrA (*40*) and VIM-2 (*41*). IMP-6 has two domains: the N-terminal domain contains four α-helices and six antiparallel β-strands and the C-terminal domain consists of two α-helices and five antiparallel β-strands (Fig. 1C). The active site is located in the cleft between the N- and C-terminal domains. Two zinc ions (Zn1and Zn2) are located 3.39 Å apart at the bottom of the shallow cleft. This distance is similar to that observed in our IMP-1 (3.35 Å) and in *S. marcescens* IMP-1 (PDB ID 5EV6; 3.34 Å). Zn1 is tetrahedrally coordinated with His116, His118, His196 and a water molecule (wat237); Zn2 is coordinated with Asp120, Cys221, His263 and wat237. The coordination geometry of two zinc ions in IMP-6 is nearly identical to that reported for IMP-1 (*28*).

As shown in Fig. 1C, IMP-1 and IMP-6 have similar overall structures, with the main chain atoms having an RMSD of 0.15 Å. Figure 1D shows a structure-based sequence alignment of IMP-type metallo-β-lactamases, whose structures have already been elucidated; L1 is a loop connecting β2 and β3 and is involved in substrate binding (*31*). Unlike other reported crystal structures of IMP-1 that show an open conformation of the L1 loop and were obtained using different crystallization conditions (PDB IDs 1DDK and 5EV6), the loop in our IMP-6 and IMP-1 structures adopt a closed conformation. Therefore, the L1 loop in the free enzyme is flexible and its conformation is possibly affected by the surrounding molecules (Fig. 1C).

### Comparison of the structures around Gly262 in IMP-6 and Ser262 in IMP-1

**Fig. 2:**
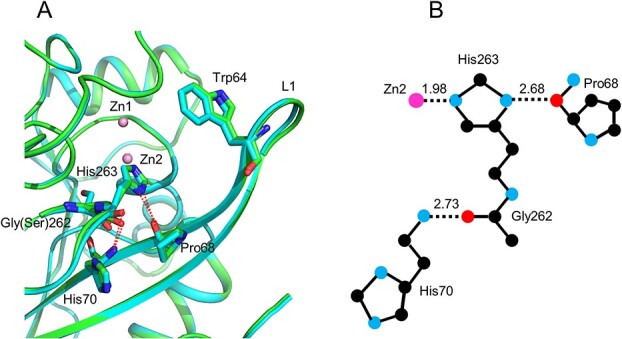
**Structural details of the region surrounding Gly262 in IMP-6/Ser262 in IMP-1.** (A) IMP-6 and IMP-1 are coloured in green and cyan, respectively. Hydrogen bonds are represented as red dot lines. Two zinc ions are represented as pink spheres. The side-chains of residues Trp64, Pro68, His70, Gly262 in IMP-6/Ser262 in IMP-1 and His263 are represented as sticks. (B) A schematic diagram depicting Gly262, HIs263 and Pro68. Hydrogen and coordinate bonds are represented as dotted lines with the respective interatomic distances.

Figure 2A shows the structural details of the region surrounding Gly262 in IMP-6 and Ser262 in IMP-1. A schematic diagram of the network of hydrogen and coordination bonds surrounding Gly262, His263 and Pro68 is shown in Fig. 2B. The carbonyl oxygen of Gly262 in IMP-6/Ser262 in IMP-1 forms a hydrogen bond with the amide nitrogen of His70 located in β3. The distances between the two atoms are 2.73 and 2.65 Å for IMP-6 and IMP-1, respectively. His263, the neighbouring residue of Gly262 in IMP-6/Ser262 in IMP-1, coordinates with Zn2 and forms hydrogen bonds with Pro68 (located in β3) and Asp120. The distances between interacting atom pairs are as follows: His263ND1 and Pro68O, 2.68 Å (IMP-6) and 2.74 Å (IMP-1); and His263NE2 and Asp120OD2, 2.83 Å (IMP-6) and 2.80 Å (IMP-1). The average displacements of Cα atoms from Ser262 to Ser264 between IMP-1 and IMP-6 were less than 0.44 Å. Therefore, the overall structure around Ser262 does not change significantly between the two enzymes.

**Table 2:** Comparison of the main chain B factors (Å ^**2**^) of the L1 loop region (residues 60–66) and the remaining regions of IMP type β-lactamases

	B factor (Å^2^)				
	IMP-1	IMP-6	IMP-2^a^	IMP-13^b^	IMP-18^c^
Loop region (x)	58.964	45.267	42.694	68.668	29.712
Other regions (y)	44.616	30.436	31.074	44.804	26.060
Ratio (x/y)	1.321	1.487	1.374	1.533	1.140

a PDB ID: 4UBQ (40).

b PDB ID: 6R78 (29).

c PDB ID: 5B3R (39).

### Comparison of the flexibility of L1

**Fig. 3:**
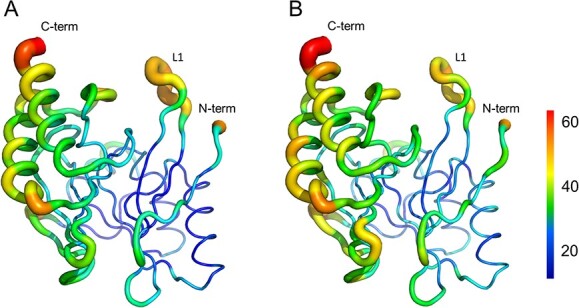
**The colour-coded main-chain trace of IMP-6 and IMP-1 according to the B factor.** (A) IMP-6. (B) IMP-1. The colour bar is indicated on the right side.

The L1 loop, also termed the flap region (*28**,**42*) or loop1 (*43*), is typical of class B1 MBLs. This region is flexible and participates in substrate/inhibitor binding (*28**,**44*). Gly262 in IMP-6/Ser262 in IMP-1 is located at the end of β11 and the main chain kinks sharply at this position (Fig. 2A). Therefore, the substitution of Ser262 with glycine will potentially increase the mobility of this turning region, especially that of His263. His263 forms a hydrogen bond with Pro68 from which L1 bends when an inhibitor/substrate is bound (*28**,**31*). Additionally, His263 moves towards the direction of Pro68 upon inhibitor/substrate binding (*28**,**31*). Thus, we expect the L1 loop of IMP-6 to be more flexible than that of IMP-1 because a glycine at position 262 should enable an easier displacement of Pro68. Analysis of the temperature factors (B factors) confirms this hypothesis. The B factor is an indicator of flexibility; it represents the degree of fluctuation of an atom in the crystal and typically decreases as the resolution increases, but the ratio between its mobile and non-mobile parts is a good indication of structural flexibility. The B factors of IMP-type β-lactamases, based on our crystal structures and those available in the Protein Data Bank, are listed in Table 2. The resolution of the IMP-6 structure is higher than that of IMP-1, which may preclude a direct comparison of B factors. However, the ratio of B factors of the loop versus other regions of the protein, which is higher for IMP-6 than for IMP-1, suggests that the L1 of IMP-6 is more flexible than that of IMP-1 (Table 2). The traces of the main chains of IMP-6 and IMP-1 are colour-coded according to their B factor value (Fig. 3). The L1 portion of IMP-6 (Fig. 3A) is thicker than that of IMP-1 (Fig. 3B), indicating that it has greater flexibility. The IMP-1 gene used in this study was from *E. coli*. The structures of *P. aeruginosa* (PDB ID 1DDK) and *S. marcesces* IMP-1 (PDB ID 5EV6) have been reported as IMP-1 from other species. The 1DDK structure is difficult to compare because of its low resolution and data quality. In the case of 5EV6, the average B factor of the loop region was 33.892 (x) while that of the non-loop region was 33.488 (y), with a ratio of 1.01 (x/y). This value is inconsistent with our results.

### Relationship between substrate affinity and B factor for IMP-type β-lactamases

In addition to IMP-1, the structures of IMP-2, IMP-13 and IMP-18 have been reported so far. The *K*_m_ values have shown that IMP-2 and IMP-13 have a high affinity for meropenem, whereas IMP-18 showed a high affinity for imipenem (Table 3) (*42**,**45**,**46*). On the other hand, the ratio of B factors of the loop and non-loop regions of the structure is higher for IMP-2 and IMP-13 than for IMP-18 (Table 2). The higher affinity of IMP-6 for meropenem is associated with the higher degree of mobility of L1, which is also observed in other IMP-type β-lactamases.

**Table 3:** Comparison of *K*_**m**_ (μM) values for imipenem and meropenem of three IMP type β-lactamases

	*K*_m_ (μM)		
Antibiotic	IMP-2^a^	IMP-13^b^	IMP-18^c^
Imipenem	24	49	9.43
Meropenem	0.3	10	13.1

a Values reported by Ricco *et al.* (42).

b Values reported by Santella *et al.* (43).

c Values reported by Softley *et al.* (39).

### Structural basis for the difference in substrate affinity of IMP-1 and IMP-6

**Fig. 4:**
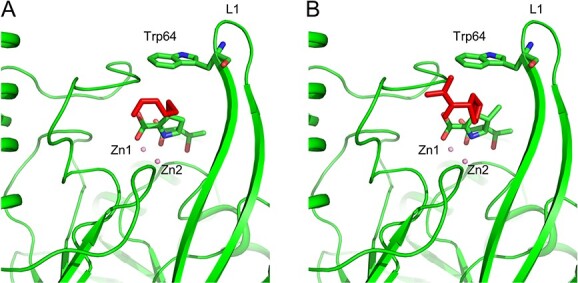
**Proposed docking models between IMP-6 and hydrolysed substrates.** (A) IMP-6 in complex with hydrolysed imipenem. (B) IMP-6 in complex with hydrolysed meropenem. The substrates and the side chain of residue Trp64 are represented as sticks. The R2 side chains of substrates are coloured in red.

The *k*_cat_/*K*_m_ ratios of IMP-1 for imipenem and meropenem are almost identical (*25**,**26*), whereas the *k*_cat_/*K*_m_ ratio of IMP-6 for meropenem is seven times higher than that for imipenem (*17*). This difference is due to the 14-fold higher *K*_m_ for imipenem in IMP-6, whereas the *K*_m_ of IMP-1 for meropenem and imipenem are nearly identical (Table 4). Assuming a Michaelis–Menten enzymatic reaction, the lower *K*_m_ indicates a higher affinity for the substrate. In other words, the affinity for imipenem is greatly reduced in IMP-6. In the present study, the structures of IMP-6 and IMP-1 were determined using crystals prepared under acidic conditions and activity measurements were performed under neutral conditions; in the case of IMP-6, we had previously submitted a structure crystallized at pH 6.5 (PDB ID 6LVJ). The main chain RMSD for both is 0.214, so there is no possibility of conformational change even under neutral conditions. In the case of IMP-1, Hinchliffe *et al.* previously submitted a crystal structure at pH 6.0 (*30*). The main chain RMSD between IMP-1 determined in this study and 5EV6 is 0.223, so there is no possibility of conformational change even under neutral conditions. Proposed docking models between IMP-6 and hydrolysed substrates are shown in Fig. 4. Meropenem and imipenem differ in the structure of their R2 side chain: the R2 of meropenem is bulkier than that of imipenem. The R2 side chains of meropenem and imipenem are oriented in the same direction and interact with the L1 loop, especially with Trp64. In IMP-6, the large flexibility of L1 facilitates the binding of meropenem to the active site and accommodates its bulky R2 side chain. Additionally, meropenem is stabilized by multiple interactions with IMP-6. These structural descriptions may explain the low *K*_m_ for meropenem in IMP-6. In contrast, imipenem, which has a smaller R2, establishes fewer interactions with the L1 loop. With fewer intermolecular interactions and a more flexible protein loop, imipenem may not be stably recognized in IMP-6, resulting in a higher *K*_m_.

**Table 4:** Kinetic parameters of wild-type and P68G mutant IMP-6 and IMP-1

	IMP-6^a^			IMP-6-P68G		
Antibiotic	*k*_cat_	*K*_m_	*k*_cat_/*K*_m_	*k*_cat_	*K*_m_	*k*_cat_/*K*_m_
	(s^−1^)	(μM)	(s^−1^·μM^−1^)	(s^−1^)	(μM)	(s^−1^·μM^−1^)
Imipenem	68	110	0.61	551	849	0.0.649
Meropenem	32	7.6	4.2	23.8	8.53	2.78
Penicillin G	51	220	0.23	141	28.5	0.495
Cephalothin	374	4.7	79.6	446	11.7	38.1
Cefotaxime	55	3.8	14.5	82.4	3.79	21.7
	IMP-1^a^			IMP-1-P68G		
Antibiotic	*k*_cat_	*K*_m_	*k*_cat_/*K*_m_	*k*_cat_	*K*_m_	*k*_cat_/*K*_m_
	(s^−1^)	(μM)	(s^−1^·μM^−1^)	(s^−1^)	(μM)	(s^−1^·μM^−1^)
Imipenem	46	39	1.2	69.0	6.92	9.97
Meropenem	44	30	1.5	8.94	18.3	0.489
Penicillin G	330	520	0.62	657	1422	0.462
Cephalothin	48	21	2.4	46.0	7.57	6.08
Cefotaxime	1.3	4	0.35	16.5	11.4	1.48

a Values reported by Yano *et al.* (15).

In the case of the IMP-13-hydrolysed imipenem complex (PDB ID 6RZR), imipenem is in three different conformations. This may be due to the weaker π–sulphur interaction with the side chain of Trp64 and the weaker hydrophobic interaction compared to meropenem (*31*). Therefore, binding interaction to L1 is reduced and affinity is decreased. The *K*_m_ for imipenem is about 5-fold higher than that for meropenem (Table 3). These results are consistent with the high L1 flexibility of IMP-13 (Table 2).

### Effect of the P68G substitution on substrate affinity of IMP-6 and IMP-1

Pro68 is located in the β3 region following the L1 loop and forms a hydrogen bond with His263. It has been reported that the L1 of IMP-1 bend at the position of Pro68 upon inhibitor binding as already described above (*28*), and a similar tendency was observed for IMP-13 upon substrate binding (*31*). In other words, Pro68 plays a key role as a hinge for L1 in IMP-1. Our discussion on substrate recognition based on the structural differences stated above suggests that L1 flexibility determines substrate preference. To further support these working models, we generated a mutant enzyme in which Pro68 is replaced with a more flexible glycine residue, measured its kinetic parameters and found the changes in substrate specificity.

The flexibility of L1 is expected to increase when Pro68 is replaced with Gly68. The kinetic constants of the P68G mutants are shown in Table 4. In IMP-1, the amino acid substitution did not substantially change the affinity for meropenem, but it increased the affinity for imipenem. When the amino acid at position 262 is a serine, the movement of His263 should not be as large as that of IMP-6; therefore, the displacement of L1 upon substrate binding to the P68G mutant may be not so different from that in the wild-type protein. This, in turn, may have increased the affinity of IMP-1 for imipenem. Materon *et al.* have shown that IMP-1 retains its hydrolytic activity upon a variety of amino acid substitutions at Pro68 (*47**,**48*). Our results are consistent with a report on IMP-18 (*42*), which also has a serine at position 262, where a T68P substitution of IMP-18 did not change the *K*_m_ for the imipenem and meropenem. In the case of IMP-6 P68G, no significant changes in *k*_cat_/*K*_m_ were observed for all substrates tested. However, while the *K*_m_ for meropenem remained unchanged, the *K*_m_ for imipenem increased by 8-fold compared to that of wild type. This suggests that the flexibility of L1 has a minor effect on the affinity for meropenem, possibly due to its bulky R2 side chain that establishes large hydrophobic interactions with L1. However, in the case of imipenem, which establishes fewer interactions with L1, the P68G substitution negatively affects affinity. These results also suggest that the glycine at position 262 in IMP-6 may promote a larger movement of His263 than Ser262 in IMP-1, and thus an increased L1 movement.

The *k*_cat_ of the IMP-6-P68G mutant for meropenem is the same as that of the wild-type IMP-6. However, the *k*_cat_ of the IMP-6-P68G mutant for imipenem is 9-fold higher than that of wild-type IMP-6 for imipenem given that the product release, preceded by the necessary L1 opening, determines the turnover rate. Because the flexibility of L1 of the IMP-6-P68G mutant is greater than that of wild-type IMP-6 and the binding of imipenem is weaker than that of meropenem, the turnover rate of imipenem by the IMP-6-P68G mutant is greater than that by wild-type IMP-6. On the other hand, the *k*_cat_ of the IMP-1-P68G mutant for imipenem is the same as that of wild-type IMP-1 for imipenem and the *k*_cat_ of IMP-1-P68G mutant for meropenem is 5-fold lower than that of wild-type IMP-1 for meropenem. In the case of the IMP-1-P68G mutant, the movement of L1 upon substrate binding to enzyme should not be very different from that in wild-type IMP-1; therefore, tight binding of meropenem may reduce the turnover rate of the mutant enzyme.

In this study, we found that the crystal structures of IMP-6 and IMP-1 are nearly identical but display different flexibilities in the L1 loop, which has a significant effect on substrate specificity. To further understand the mechanisms underlying substrate specificities in IMP-type MBLs, we are currently analysing the structures of IMP-6 and IMP-1 in complex with bound substrates.

## Funding

This work was supported by Grants-in-Aid for Scientific Research (Kiban C, no. 17K09018 and no. 21K07010) from the Japan Society for the Promotion of Sciences.

## Conflict of Interest

None declared.

## Data availability

The coordinate and structure factor files from this study have been deposited in the Protein Data Bank under the accession numbers 7XHW (IMP-1) and 7XHX (IMP-6).
